# Video Remote Interpreting Technology in Health Care: Cross-Sectional Study of Deaf Patients’ Experiences

**DOI:** 10.2196/13233

**Published:** 2019-03-11

**Authors:** Poorna Kushalnagar, Raylene Paludneviciene, Raja Kushalnagar

**Affiliations:** 1 Department of Psychology Gallaudet University Washington, DC United States; 2 Department of Science, Mathematics, and Technology Gallaudet University Washington, DC United States

**Keywords:** video remote interpreting, deaf, sign language, assistive technology, accessibility, communication

## Abstract

**Background:**

The advent of new rehabilitation and assistive technologies has led to the creation of video remote interpreting (VRI) as an accessible communication technology for deaf patients. Although there has been a rapid growth in the use of VRI technology by health care providers, there is scant published information on VRI users and their satisfaction. Current, timely data are needed to understand deaf patients’ use and satisfaction with the quality of VRI technology in health care settings.

**Objective:**

This study aimed to investigate the national trends of deaf patients’ satisfaction with the quality of video remote interpreting (VRI) in health settings and recommend actions to improve VRI quality and deaf patients’ satisfaction with VRI in health care settings.

**Methods:**

Secondary data related to deaf adults’ experiences of using VRI service in a medical setting were obtained from the Health Information National Trends Survey in American Sign Language, which was administered to a US sample of deaf adults between 2016 and 2018.

**Results:**

Among our VRI users (N=555, all in the United States) who answered questions about VRI usage in health between 2016 and 2018, only 41% were satisfied with the quality of the VRI technology service. Respondents with fewer years of education or those who were male were more likely to rate the VRI quality as acceptable. After adjusting for covariates in a binary regression analysis, deaf patients’ self-reported interference (ie, VRI interpreter’s interference with disclosure of health information) increased patient dissatisfaction with the quality of VRI technology service by three-fold.

**Conclusions:**

To increase satisfaction with VRI technology service in health care and rehabilitation settings among deaf patients, special attention needs to be given to video technology, as the use of sign language requires high-fidelity video for optimal communication between the interpreter and patient. To promote the willingness to disclose medical information through VRI among deaf patients, the interpreter must be highly skilled in both expressive and receptive communication and have the requisite background in medicine and rehabilitation.

## Introduction

Around 500,000 people are deaf or hard of hearing (termed as “deaf” henceforth) in the United States and rely primarily on American sign language (ASL), which requires visual communication [1]. As such, they have much in common with members of other linguistic and cultural minority groups, due to their reliance on ASL over English for daily communication. For this reason, among others, ASL users continue to report difficulties accessing health care many years after passage of the Americans with Disabilities Act of 1990. Many rely on in-person ASL interpreters for effective communication with health care or rehabilitation professionals, but still report difficulties accessing health care due to geographical, time, and financial constraints in booking ASL interpreters. A solution to these constraints is the advent of video remote interpreting (VRI) technology, which is a specialized translation service that relies on a high-speed internet connection and a camera-equipped device to connect a remote interpreter with a health provider and a patient to facilitate their communication [2,3]. VRI technology is not subject to geographical and time constraints, since the interpreter can provide services from anywhere and does not have to spend time commuting to and from the appointment.

VRI is often used for sessions, one-on-one visits, and patient walk-ins when an interpreter is needed immediately. The equipment for VRI typically consists of a tablet that is sometimes mounted on a rolling stand that can be moved around, with an adjustable position and location. The medical provider is responsible for providing the tablet with the interpreter, which is brought in when the deaf patient is present. The medical provider then presses a button on the tablet to connect to an interpreter stationed elsewhere. This video connection depends on the internet and is usually wireless. The interpreter is portrayed on the whole screen, and the video of the patient is shown in a smaller box in the corner of the screen.

However, in practice, VRI equipment has significant limitations compared to on-site interpreting service for the health care provider, patient, and interpreter in terms of interaction and visibility. For example, while an on-site interpreter can independently move and focus on either the deaf person or health care provider, the tablet video is usually focused on the deaf patient, and consequently, the VRI interpreter cannot see the body language and gestures of the health care provider when they are talking. Similarly, on-site interpreters are in a better position to filter background audio or focus on multiple speakers, while VRI interpreters experience more challenges in filtering noises and attending to key messages. Finally, VRI services are prone to more technical and logistical barriers due to the lack of familiarity regarding their use by health care providers and deaf users.

Because VRI technology is new, empirical research is still emerging and, to date, only one published qualitative study was performed outside the United States. Much of the relevant literature has focused on lawsuits and complaints, which are outside the scope of this paper. In a qualitative study of 58 interpreters, about half of the sample had a positive experience with VRI, primarily because they could work from home and immediately provide accessibility when called [4]. The negative experiences reported by the other half were due to the poor quality of video technology, low bandwidth, and issues arising from the limited range of visual cues in the environment. This study did not include experiences or perspectives of deaf patients who used VRI. Although there has been a rapid growth in the use of VRI by health care and rehabilitation providers, current data are needed to understand deaf patients’ experiences with VRI technology. This study investigates the trends of deaf patients’ use of and satisfaction with the quality of VRI technology service in health settings.

## Methods

### Materials and Data Source

With approval from the institution’s human subjects review board and informed consent from the participants, data related to deaf adults’ experiences of using VRI service in a medical setting were obtained from the Health Information National Trends Survey in ASL, which was administered to a US sample of deaf adults between 2016 and 2018 [5]. The VRI items were drafted and revised by a team of deaf experts with extensive experience using this technology in health care. These items were translated and back translated by deaf bilingual professionals. The translated items were then tested for clarity and understanding through cognitive interviews with deaf people who had a high school or less education [5]. The final translated items were then filmed and uploaded to an online survey platform prior to administration. All items had ASL videos with English text.

### Responses

This paper focuses on the responses to the following three questions directly related to patients’ opinions and experiences with VRI.

#### Interpreter Choice

Participants were asked, “If you had to choose one, how do you prefer to use an interpreter in health settings?” with three response options provided: “On-site,” “Through video remote interpreting,” and “Doesn’t matter.”

#### Quality Rating of the Video Remote Interpreting Service

Participants were asked, “How would you rate the quality of VRI services you received in healthcare settings in the past 12 months?” with six response options provided: “Excellent,” “Very good,” “Good,” “Fair,” “Poor,” and “Did not use VRI.” In the analysis, responses of “Excellent” to “Good” were recoded as *Satisfactory* and “Fair” to “Poor” were recoded as *Unsatisfactory*.

#### Disclosure of Health Information in Front of a Video Remote Interpreting Interpreter

Participants were asked, “Do you feel having a VRI will interfere with your disclosure of health information with the doctor?” with two response options provided: “Yes” and “No.”

### Participant Recruitment, Consent, and Other Study Procedures

Following institutional review board approval, the research staff began recruitment through national channels, focusing on ASL-using deaf community members. Given the nature of this low-incidence, hard-to-reach population, a purposive strategic respondent-driven sampling method was used to ensure adequate inclusion of deaf signers across the United States. Recruitment methods included snowball and respondent-driven samplings that were found to be effective for deaf and hidden populations [6,7], flyers, and advertisements on deaf-centered organizations’ websites and electronic newsletters. Bias associated with snowball sampling was overcome with a large sample size [8]. Communication occurred through accessible channels, including mail, email, social media, and videoconference programs. Prospective participants were informed that the survey included questions about health status, health communication, and health behaviors.

Inclusion criteria were use of ASL as a primary language, age of 18 years or above, and presence of bilateral hearing loss. Each participant received a gift card for participating in the study. The survey took approximately 1 hour to complete. No names or identifying information was included in the online survey, and a unique identifier was used to avoid storing personal information in the same online survey dataset. The identifying information was stored in a separate database that was accessible only to the principal investigator.

### Statistical Analyses

Descriptive statistics were used to summarize the sociodemographic and health care accessibility sample characteristics of deaf individuals who used VRI in health care settings within the past 12 months. Unweighted descriptive statistics, such as cross-tabulation and percentage procedures, were used to describe the sample. Binary logistic analysis was used to predict the odds of reporting satisfaction with the quality of VRI services, after controlling for sociodemographic covariates.

## Results

### Sample Characteristics of Video Remote Interpreting Service Users

Of the 968 deaf adults who answered questions related to the use of VRI in health care, 413 never used VRI within the past 12 months and were excluded from analyses. The focus of this study was on participants who have actually used VRI in the past year and were able to provide their perspectives on the direct firsthand experience of using VRI. The final VRI user sample (N=555; mean age 45 years, SD 18 years) included 37% persons of color and 30% respondents who self-identified as sexual/gender minority. Although just over half of the sample had a job, 46% percent had a college degree and 43% fell in the middle-income category. Over 90% had insurance, including Medicare/Medicaid and private insurance, and about 88% rated their health as good, very good, or excellent. When asked how much one could understand (listening, speechreading, or both) a hearing person in a quiet room, about 25% of the sample could not understand at all and another 25% self-rated their listening or speech-reading ability as high.

### Quality of the Video Remote Interpreting Service According to Video Remote Interpreting Users

Users’ satisfaction with the VRI service quality according to the sociodemographic variables is presented in Table 1. About 41% (n=228) of the deaf patient sample rated the quality of VRI as satisfactory. The rest (n=327, 59%) rated their VRI experience as unsatisfactory. Results suggest that male gender or high school education has a greater influence on satisfaction of VRI service quality than of dissatisfaction.

With regard to health care accessibility indicators (Table 2), respondents who had a health care provider that they saw regularly were significantly more likely to be dissatisfied with the quality of VRI service compared to respondents who did not have a regular provider (Χ^2^=7.0; *P=*.011). Deaf patients who reported that VRI interfered with disclosure of health information to their health care provider were less likely to be satisfied with the quality of VRI service (Χ^2^=47.2; *P*<.001).

A model-building approach was used to determine the best fit. In the first model, all sociodemographic and health indicators were included in the analysis. Significant (*P*<.05) and nominally significant (*P*<.10) variables from the first model were retained for evaluation in the next model. Noncontributing variables that were not significant were removed, and the model was evaluated for significance. This procedure was repeated for the third model. The model that had the largest likelihood value was the final chosen model, with VRI service quality as an outcome (Χ^2^=32.3,  *P*<.001). This model with six variables explained 12% (Nagelkerke  *R*^*2*^) of the variation in VRI service quality rating and correctly classified 64% of cases. Presence of a regular provider and VRI interference (with health information disclosure) were significantly associated with deaf patients’ ratings of the VRI service quality (Table 3). Respondents who did not have a health care provider that they saw regularly were 1.5 times more likely to rate the VRI service quality as satisfactory as compared to respondents who had a regular provider. Moreover, those who felt that VRI did not interfere with disclosure of health information were three times more likely to report satisfaction with VRI service quality.

**Table 1:** Sociodemographic characteristics of users with regard to satisfaction with the video remote interpreting service quality in health care settings (N=555). Frequencies that do not add up to the total sample size reflect missing responses.

Characteristics		Satisfied with VRI^a^ service quality (n=228)	Not satisfied with VRI service quality (n=327)	Chi-square value
Age (years), mean (SD)		46 (19)	44 (17)	0.8^b,c^
**Gender, n (%)**				5.0^c^
	Male	114 (50.2)	129 (40.6)	
	Female	113 (49.8)	189 (59.4)	
**Race/ethnicity, n (%)**				2.4
	White	134 (59.0)	214 (65.4)	
	Non-white	93 (41.0)	113 (34.6)	
**Education, n (%)**				7.4^c^
	High school	77 (34.5)	80 (24.7)	
	Some college	52 (23.3)	74 (22.8)	
	College	94 (42.2)	170 (52.5)	
**Occupation, n (%)**				2.7
	Employed	117 (51.3)	182 (56.0)	
	Student	25 (11.0)	36 (11.1)	
	Retired	48 (21.1)	51 (15.7)	
	Unemployed	38 (16.7)	56 (17.2)	
**Income** **, n (%)**				0.6
	Lower	98 (44.1)	152 (47.4)	
	Middle	100 (45.0)	138 (43.0)	
	Upper	24 (10.8)	31 (9.7)	
**Region, n (%)**				2.5
	Northeast	18 (7.9)	34 (10.4)	
	South	95 (41.7)	123 (37.6)	
	Midwest	44 (19.3)	75 (22.9)	
	West	71 (31.1)	95 (29.1)	
**Health insurance, n (%)**				1.1
	Yes	212 (96.4)	302 (94.4)	
	No/not sure	8 (3.6)	18 (5.6)	
**General health, n (%)**				3.9
	Excellent/very good	125 (55.1)	153 (46.9)	
	Good	80 (35.2)	130 (39.9)	
	Fair/poor	22 (9.7)	43 (13.2)	

^a^VRI: video remote interpreting.

^b^*t* value.

^c^*P*<.05.

**Table 2:** Health care access characteristics with regard to satisfaction with video remote interpreting quality in health care settings within the past year (N=555). Percentages are determined by the total number of responses to each question.

Characteristics		Satisfied with VRI^a^ quality (n=228), n (%)	Not satisfied with VRI quality (n=327), n (%)	Chi-square value
**Regular provider**				7.0^c^
	Yes	113 (50.4)	201 (61.8)	
	No	111 (49.6)	124 (38.2)	
**Frequency of visits to regular provider**				5.3
	Never	36 (16.7)	30 (10.0)	
	A few times	133 (61.9)	208 (68.1)	
	Many times	46 (21.4)	66 (21.9)	
**Hospital admission**				0.01
	Yes	32 (26.2)	46 (26.4)	
	No	90 (73.8)	128 (73.6)	
**Emergency room visit**				0.2
	Yes	47 (38.5)	72 (41.1)	
	No	75 (61.5)	103 (58.9)	
**VRI interpreter presence interfering with disclosure of health information to the doctor**				32.7^d^
	Yes	171 (75.0)	166 (50.9)	
	No	57 (25.0)	160 (49.1)	

^a^VRI: video remote interpreting.

^c^*P*=.011.

^d^*P*<.001.

**Table 3:** Logistic regression results for satisfaction with the quality of the video remote interpreting service (reference group: patients not satisfied).

Variable	Adjusted odds ratio (95% CI)	*P* value
Age	1.01 (0.99-1.02)	.19
Education^a^	1.36 (0.94-1.96)	.10
Gender^b^	0.73 (0.51-1.05)	.09
Race^c^	1.30 (0.88-1.91)	.16
Regular provider^d^	1.50 (1.04-2.17)	.03
Interpreter interference^e^	2.90 (1.97-4.27)	<.001

^a^Reference group: Patients with a college degree.

^b^Reference group: Male patients.

^c^Reference group: White patients.

^d^Reference group: Patients responding “Yes.”

^e^Reference group: Patients responding “Yes.”

## Discussion

### Overview

Our study of patient-reported outcomes is the first to report US findings related to deaf patients’ experience with VRI technology. Rigorous data-collection approaches were used to ensure that the sample was inclusive of diverse members in the deaf community that use ASL. Our study results suggest that over half of the participants do not find the quality of VRI services to be satisfactory, despite regulations that specify minimum quality of standards for both technology and interpreter qualifications. Our study also showed that VRI interference with health information disclosure is a crucial variable for satisfaction with the quality of VRI service among deaf patients. Further research is needed to clarify whether VRI interference is affected by the use of an interpreter or video technology itself.

### Advantages and Disadvantages of Video Remote Interpreting Technology

Below, we discuss the advantages and disadvantages of VRI that might have affected deaf patients’ responses in our study and conclude with recommendations to rectify the VRI interference with deaf patients’ disclosure of health information and to increase their satisfaction with the quality of VRI service.

#### Advantages

There has been a rapid adoption and use of VRI as the first choice to support accessible and effective physician-provider communication in health care. Health care and rehabilitation providers may choose to provide VRI over traditional in-person interpreters due to the former’s cost and flexibility.

VRI tends to be cost effective, as VRI interpreters are reimbursed only for the short amount of time that they are required for (eg, 15 minutes), and there is no need to preschedule, which means no cancellation fees. There is usually a minimum time cost for in-person interpreters [9]. For a 20-minute appointment with a deaf patient, the provider is billed 2 hours for an in-person interpreter. In addition, in emergency room or patient situations, in-person interpreters would often be present throughout the entire stay, while the VRI can be connected and disconnected on an as-needed basis when communication needs arise.

VRI offers more flexibility in terms of scheduling, as it takes a variable amount of time for an in-person interpreter to travel to the meeting site. In emergency situations, VRI can quickly assist with communication, while an in-person interpeter would need to travel to the site to provide communication access [10]. VRI has a wider geographical reach and offers access to a larger pool of interpreters including interpreters who have experience in medical settings and specialized training in medical interpreting [11]. The use of qualified interpreters can reduce the possibility of miscommunication between the medical care provider and the patient.

#### Disadvantages and Recommended Improvements

In most health care settings, VRI is usually an add-on on-call service and considered to be an alternative to the in-person interpreting service. Such an assumption can lead to the emergence of technical problems such as slow connections or limited bandwidth, which impedes effective communication. For example, VRI needs to be free of blurriness, freezing, and connectivity issues. Since VRI usually relies on wireless connections, which are subject to interference, the quality of video can be suboptimal. Effective sign language communication requires both clear and uninterrupted video and qualified interpreters. When the video quality is not optimal, the quality of patient-provider communication is impacted and affects the accuracy of the translation and relay of the deaf patient’s health information to the health care provider. When the message is misunderstood or gets lost in the translation, it impacts the deaf patient’s satisfaction with VRI services. Conversely, when the video quality is clear, the interpreter’s expressive and receptive language skills must be highly proficient in order to support effective communication that takes place between the deaf patient and health care provider.

The combination of effective VRI technology and highly qualified interpreters allows patient-centered care to take place. When a deaf patient experiences positive patient-centered care, it increases patient-provider trust and patient outcomes [12,13]. These have strong potentials to reduce health disparities among medically underserved groups of deaf patients, including reduction of mortality from life-threatening diseases, improved management of chronic diseases, better understanding of treatment plans, and higher self-efficacy of adherence to medications.

The set-up time can also impact deaf patients’ satisfaction with the VRI service. When the VRI system is quickly set up and connected to a call center that employs interpreters with strong receptive and expressive skills, the wait time will be shorter [14]. If the patient is seen quickly and provided with a fully functioning VRI system with qualified interpreters, this system can potentially reduce the number of emergency visits and unnecessary diagnostic tests, all of which are associated with cost burden.

### Future Research: Evaluation of Certified Deaf Interpreters to Improve Communication Through the Video Remote Interpreting Service

VRI interferes with the health information disclosure possibly through communication difficulties between the deaf patient and interpreter, which needs to be evaluated in a future study. Selecting a certified deaf interpreter via VRI, who is usually listed as a “deaf interpreter” instead of an “ASL interpreter” on the list of languages on the VRI, can potentially resolve the communication problems and decrease the feelings of VRI interference with disclosing health information. Certified deaf interpreters are deaf people who work as professional interpreters, often acting as an intermediary between the interpreter who can hear (hearing ASL interpreters) and the deaf client.

Certified deaf interpreters are in a unique position to help improve the quality of the patient-physician interaction even when VRI is used. For example, they are very perceptive to body language and subtle changes in facial expressions and sensitive to cultural issues that may impede communication between the medical provider and the deaf patient [15]. They can also reduce the impact of technical issues that modify language use [16]. Examples of technical problems that modify sign language use include the limited viewing angle of the tablet with VRI and limited ability to follow focus of the conversation. When this occurs, interpreters and deaf patients may have the tendency to simplify their signs to deal with these constraints, which can affect the quality of the patient-physician interaction.

A small-scale study on spoken-language VRI services found that spoken-language interpreters were adapting to the new VRI technology used by foreign patient speakers [17]. Therefore, it is possible that CDIs have more experience with adapting to the constraints associated with VRI technology angles and are able to fill in missing contexts that were affected by the modification in sign-language use. Future research should consider assessing the role of CDI in reducing the constraints associated with VRI technology angles, increasing the efficiency of communication between the medical provider and the deaf patient, and ultimately increasing the deaf patient’s trust in the provider.

### Limitations

Although we asked for deaf respondents’ preference between on-site interpreter and VRI, we did not inquire whether they chose to experience VRI or were forced to do so due to various reasons. Deaf patients are often presented with VRI technology or an in-person interpreter when they show up at an appointment, and it is difficult to switch to a preferred method of communication at the last minute. If a majority of participants were forced to use VRI, it might have contributed to the low preference scores in this study.

### Conclusions

To increase satisfaction with VRI technology and service in health care and rehabilitation settings, special attention needs to be given to the video quality and customer control of VRI, as sign-language communication requires high-fidelity video for the patient be able to understand the interpreter and vice versa. To promote the deaf person’s willingness to disclose medical information to the provider and increase trust in patient-physician communication, the interpreter must be highly skilled in both expressive and receptive communication and have the requisite background in medicine and rehabilitation.

## Abbreviations

VRI: video remote interpreting
ASL: American Sign Language
